# Monkeypox and occupational exposure: Potential risk toward healthcare workers and recommended actions

**DOI:** 10.3389/fpubh.2022.1023789

**Published:** 2022-09-26

**Authors:** Ranjit Sah, Aroop Mohanty, Parul Singh, Abdelaziz Abdelaal, Bijaya Kumar Padhi

**Affiliations:** ^1^Tribhuvan University Teaching Hospital, Institute of Medicine, Kathmandu, Nepal; ^2^Harvard Medical School, Boston, MA, United States; ^3^Department of Microbiology, All India Institute of Medical Sciences, Gorakhpur, India; ^4^Bachelor of Medicine, Bachelor of Surgery, Faculty of Medicine, Tanta University, Tanta, Egypt; ^5^Department of Community Medicine and School of Public Health, Postgraduate Institute of Medical Education and Research, Chandigarh, India

**Keywords:** Monkeypox, occupational exposure, healthcare workers (HCWs), healthcare (MeSH), occupational hazard

Monkeypox virus (MPXV) is the microorganism responsible for causing the zoonotic disease known as Monkeypox (MPX). It is a double-stranded DNA virus belonging to the Orthopoxvirus genus of the Poxviridae family (1). Other important viruses of this family include the variola virus which causes the smallpox disease and the vaccinia virus which is utilized to manufacture the smallpox vaccine. The first case of human MPX was seen in 1970 in a pediatric patient in the Democratic Republic of Congo (DPR). Since then, the disease was limited to the African continent with only a few sporadic outbreaks outside of endemic regions. However, during the recent multi-country outbreak of MPX, more than 89 countries have been affected, and the majority of them are reporting MPX cases for the first time (2, 3).

Although human-to-human transmission of the disease has become more evident in the recent outbreak, the risk of occupational exposure, particularly in healthcare settings, should be carefully addressed. A recent review highlighted that there is a low risk of exposure to MPX in non-endemic healthcare settings; however, the level of evidence remains limited (4). A dreaded picture was seen during the COVID-19 pandemic when more than 115,000 healthcare workers (HCWs) lost their life due to this fatal disease (5). Important to say, HCWs constituted a significant proportion of all COVID-19 patients. Similarly, in the present MPX outbreak, the nosocomial transmission of MXPV can occur, potentially leading to the deaths of HCWs, which can, subsequently, turn this disease into an occupational hazard.

Concerns regarding MPX transmission in medical settings arose from reports of person-to-person transmission during this multi-country outbreak and the long-standing evidence of smallpox transmission in hospitals (6–9). On 17th August 2022, a total of 35,275 confirmed cases of MPX have been reported, out of which 3923 HCWs were suspected of MPX and 386 tested positive for MPXV (10). However, the majority of these HCWs were infected in the community rather than hospital settings with 98.3% falling under the MSM (men who have sex with men) community. For instance, in a case reported in North London on 11th June 2022, a 35-year-old, HIV-positive, male, sexual HCW presented with fever, lymph node swelling, myalgia, and throat pain (11). Subsequently, he developed a painful blister on his nose which gradually increased in size. He tested positive for MPXV and was treated with Tecovirimat (antiviral agent licensed by the European medicine agency) for 10 days, after which he was discharged.

Further investigations are still ongoing to determine the possible routes of transmission of Monkeypox among other three HCWs who were reported at the same time but had no history of sexual contact. With the progressive increase in the number of MPX cases worldwide, HCWs are now at increased risk for contracting the disease. When caring for a patient who has MPX, the proper and consistent use of personal protective equipment (PPE) is extremely important in protecting, and preventing the transfer of MPXV to HCWs. Importantly, HCWs are not limited to physicians and nurses; however, they rather include emergency medical technicians, nursing assistants, technicians, therapists, phlebotomists, pharmacists, students, trainees, and contractual workers who are not employed by the healthcare facility, and those not directly involved in patient care. In addition, HCWs involve those who are exposed to infectious pathogens that can be transmitted in different sectors within healthcare settings, such as laundry, security, engineering and facilities management, administrative, billing, and volunteer personnel. Unaddressed mistakes (i.e., self-contamination following the removal of contaminated PPE) could pose a significant risk of transmission to HCWs.

In the study of Fleischauer et al. (12), nearly 75% of exposed HCWs reported at least one unprotected exposure to a confirmed MPX patient. According to postexposure surveillance, none of the HCWs reported any symptoms which were in line with the case definition for MPX infection (12).

However, HCWs should be aware of the signs and symptoms of MPX while entering a contaminated patient room or a treatment center, and they should wear the recommended PPE. If any of these symptoms occur, they should contact health services for further evaluation and should not report to work (or should leave work, if signs or symptoms develop while at work). Then, authorized officials and public health authorities should decide how to monitor exposed HCWs. In general, the monitoring strategy used usually reflects the risk of transmission, with more active-monitoring techniques being used for exposures in higher-risk settings. In most cases, self-monitoring techniques are adequate for exposures with low transmission risks. If authorized officials and public health authorities decide that a self-monitoring technique is appropriate, even greater risk exposures might be covered. The sort of monitoring to be employed is ultimately determined by the individual's level of exposure risk, their dependability in reporting potential symptoms, the number of people who need monitoring, the amount of time since exposure, and whether or not they have received postexposure prophylaxis (PEP) (13–15).

According to the Centers for Disease Control and Prevention (CDC), asymptomatic HCWs who have been exposed to MPXV do not need to be isolated and prohibited from working, but they should be screened for symptoms during the 21 days following their last exposure. If symptoms appear, HCWs should be treated. If a diagnosis that calls for a work restriction is made, even after MPX infection has been ruled out, there may still be limits advised (e.g., varicella). Throughout the 21-day observational period, if HCWs develop a rash, they should remain off work until the rash is examined and confirmatory testing is carried out to either confirm or exclude a diagnosis of MPX (13–15).

A new 5-day isolation phase should be initiated if a new symptom appears without any rash at any point throughout the 21-day monitoring period, and the HCWs should be restricted from working. Even if the 5-day period lasts longer than the original 21-day monitoring period, HCWs should be restricted from work for 5 days after the onset of any new symptom if there is no rash. HCWs may return to work with approval from their workplace officials after the 5 days have elapsed without the onset of any new symptoms and a complete skin examination confirms no skin abnormalities. Until all lesions have crusted, those crusts have dissolved, and a new layer of healthy skin has grown underneath, HCWs with a confirmed MPX infection should stay off work. The exact time interval for which an HCWs can resume work will ultimately be decided by public health authorities (13–15). The rising signals on MPX leading to a global public health concern along with COVID-19. We will gain more clarity on the magnitude of the current outbreak as case finding intensifies. Protecting HCWs and ensuring that we learn from recent epidemics and share available resources early, and quickly will be the key to containing the transmission.

## Author contributions

RS: write the initial draft. AM, PS, AA, and BP: review the literature and edit the manuscript. All authors agree for the final manuscript.

## Conflict of interest

The authors declare that the research was conducted in the absence of any commercial or financial relationships that could be construed as a potential conflict of interest.

## Publisher's note

All claims expressed in this article are solely those of the authors and do not necessarily represent those of their affiliated organizations, or those of the publisher, the editors and the reviewers. Any product that may be evaluated in this article, or claim that may be made by its manufacturer, is not guaranteed or endorsed by the publisher.
